# Right ventricular T1 mapping is technically feasible and correlates with right ventricular dysfunction in non-ischemic cardiomyopathy

**DOI:** 10.1186/1532-429X-16-S1-P336

**Published:** 2014-01-16

**Authors:** Christine Jellis, Teerapat Yingchoncharoen, Ayman Ayache, Scott Flamm, Deborah Kwon

**Affiliations:** 1 Cardiovascular Medicine, Cleveland Clinic, Cleveland, Ohio, USA; 2 Diagnostic Radiology, Cleveland Clinic, Cleveland, Ohio, USA

## Background

T1 mapping is increasingly employed for evaluation of diffuse fibrosis within the left ventricular (LV) myocardium. Physiological confounders require correction of raw data prior to analysis. T1 values have been inversely correlated with LV systolic and diastolic function. We sought to evaluate the feasibility of right ventricular (RV) T1 mapping and to assess its relationship with RV function.

## Methods

Post-contrast, T1 mapping was performed with a Look-Locker technique using inversion recovery imaging in 102 consecutive subjects with non-ischemic cardiomyopathy. Regional T1 values of the RV free wall, RV septum and lateral LV were calculated using prototype analysis software. CMR volumetric data was measured offline using a cine short axis stack to establish RV end-diastolic-volume (RVEDV), end-systolic-volume (RVESV) and ejection fraction (RVEF). Simultaneous subject biochemical and anthropometric data were recorded.

## Results

Subjects demonstrated mild to moderately impaired global RV systolic function (RVEF = 37 ± 11%; RVEDV = 212 ± 74 ml; RVESV = 140 ± 73 ml). LV function was moderately reduced (LVEF 31 ± 17%). Good correlation was observed between mean LV and RV T1 values [Table 1], with similar T1 values maintained in both the RV free wall and septum (r = 0.601, p < 0.001). RV free wall T1 values demonstrated an expected correlation with heart rate (HR) (r = -0.253, p = 0.010) but not other known covariates of LV T1 values (renal function, weight or contrast factors). Upon stratification according to RV free wall T1 value (Group 1 T1 < 310 ms; Group 2 T1 > 310 ms), RVEF was proportionally related to T1 (Group 1 mean RVEF 30 ± 10%, Group 2 mean RVEF 39 ± 10%; p = 0.011).

**Table 1:** 

Correlation between LV and RV T1 values (mean T1 value)	RV free wall T1 value (350 ± 66 ms)		RV septal T1 value (359 ± 74 ms)	
	**r**	**p**	**r**	**p**

Lateral LV T1 value (384 ± 78 ms)	0.624	< 0.001	0.646	< 0.001

## Conclusions

Right ventricular T1 mapping appears technically feasible with good agreement between regional RV and LV T1 values. RV T1 values appear proportionally related to RV ejection fraction, suggestive that RV dysfunction in this population may be mediated by fibrotic factors.

## Funding

No funding disclosures.

